# Global research trends and hotspots in calcaneal fracture: A bibliometric analysis (2000–2021)

**DOI:** 10.3389/fsurg.2022.940432

**Published:** 2023-01-06

**Authors:** Yang-Ting Cai, Yu-Ke Song, Min-Cong He, Xiao-Ming He, Qiu-Shi Wei, Wei He

**Affiliations:** ^1^ The Third Affiliated Hospital of Guangzhou University of Chinese Medicine, Guangzhou, China; ^2^ Guangdong Research Institute for Orthopedics and Traumatology of Chinese Medicine, Guangzhou, China; ^3^ Guangzhou University of Chinese Medicine, Guangzhou, China

**Keywords:** calcaneal fractures, bibliometric analysis, visualization, research trends, hotspots

## Abstract

**Background:**

Calcaneal fracture is common and carries high morbidity and disability. Its treatment is therefore of vital concern. Many topics concerning calcaneal fracture remain controversial, and the subject has not yet been well-researched. We aim to analyze and illustrate the trends in development, overall knowledge structure, “hotspots,” and research frontiers on the topic of calcaneal fracture.

**Methods:**

Literature relating to calcaneal fracture published between 2000 and 2021 was retrieved from Science Citation Index Expanded (SCIE) database of the Web of Science. Three bibliometric tools (Bibliometrix, CiteSpace, and VOSviewer software) were used for analysis and the generation of knowledge maps. Annual trends in publication counts and the relative contributions of different countries, regions, institutions, authors, and journals, as well as keyword clusters, “hot topics,” and research frontiers, were analyzed.

**Results:**

A total of 1,687 publications were included in the analysis. The number of calcaneal fracture articles published worldwide each year was highest in 2019, with a total of 128 articles. The United States has made the greatest contribution to the field, with the largest number of publications and the highest H-index. *Foot & Ankle International* was the most productive journal, publishing a total of 167 articles on calcaneal fracture during the study period. Hebei Medical University of China and the University of California, San Francisco were the most prolific institutions. Professors T. Schepers, S. Rammelt, H. Zwipp, and Y. Z. Zhang have made remarkable contributions to the field. However, the degree of collaboration between researchers and among institutions was relatively low, and took place mainly in Europe and the Americas. All relevant keywords could be categorized into three clusters: studies of internal fixation, studies of fractures, and studies of osteoporosis. A trend of balanced and diversified development could be seen within these clusters. Keywords with ongoing “citation bursts,” such as sinus tarsi approach, wound complications, minimally invasive technique, extensile lateral approach, surgical treatment, and plate, may continue to be research “hotspots” in the near future.

**Conclusion:**

Based on current global trends, the number of publications on calcaneal fracture will continue to increase. Topics such as minimally invasive techniques and complications have become important hotspots of research. We recommend enhancing international communication and collaboration for future research in this field.

## Introduction

Calcaneal fracture is the most common fracture of the tarsal bones, constituting 65% of all tarsal fractures and 1%–2% of fractures overall (1). Because of its complex anatomy and physiology, the significant degree of associated trauma, and the difficulty it presents in terms of surgical repair, calcaneal fracture is frequently associated with poor surgical prognosis and many sequelae; its disability rate is as high as 30% (2). The best practices for calcaneal fracture management have therefore generated much discussion and controversy over the past two decades; to this day, interest in the subject, especially as a topic of research, remains high. Some prospective randomized controlled trials have indicated that surgical treatment has no significant advantages compared with conservative care (3, 4). Although traditional approaches using an extended L-shaped lateral incision with lateral plating can offer good fracture visualization and direct reduction, they also have high rates of wound complications and infection—up to 37% and 20%, respectively (5). In recent years, minimally invasive techniques have been developed, including limited-incision sinus tarsi approach and internal fixation, percutaneous fixation, and arthroscopic-assisted fixation (6). These emerging techniques may be beneficial for patients with lower complication rates. Despite intense research efforts and advances in diagnostic and therapeutic strategies, many open questions remain, especially on the topics of direct and indirect costs and disability, and the consequences of calcaneal fracture thus continue to take a toll on society. Moreover, the rapid growth of publications on calcaneal fracture makes it difficult for researchers to identify relevant information and keep track of new developments and research directions in this field. Thus, it is necessary to take stock of the global status of calcaneal fracture research.

Bibliometric analysis is a new interdisciplinary research method that integrates statistics and visualization techniques, and it is frequently used in orthopedic surgery (7). It helps researchers understand the trends and patterns in specific topics, including the current research “hotspots,” within a particular domain (8). It is widely used in research that focuses on the analysis of specific diseases, as it provides essential information for further research on their prevention and treatment (9–12). However, no bibliometric study of calcaneal fracture research has yet been published. Therefore, this study sought to use bibliometrics to analyze the field of calcaneal fracture (i.e., the overall research framework, development trends, and research hotspots) for the first time. In addition, we wanted to provide scientific researchers with more information about the field’s current status and trends. We hope that future scholars will continue to build upon this preliminary research.

## Methods

### Data source

The data for this study come from the Web of Science Core Collection (WOSCC). Web of Science, a world-renowned database containing many influential and high-quality journals, has been widely used in bibliometric research.

### Retrieval strategies and data extraction

**Figure 1:**
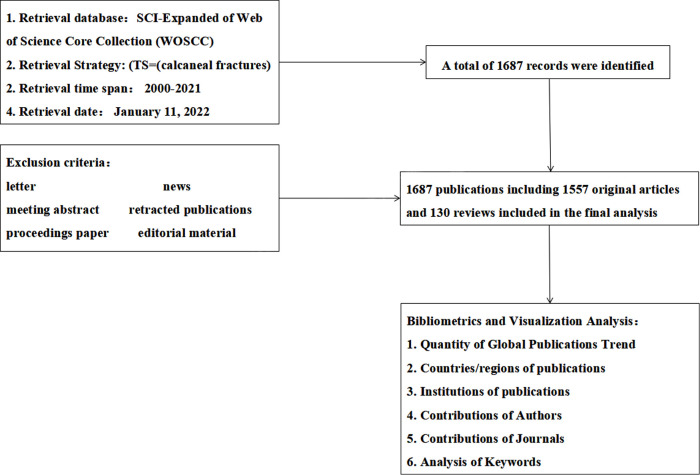
Flowchart describing the selection process for articles and reviews included in the study.

The search strategy was (TS = calcaneal fractures). The included publication types were limited to original articles and reviews; meeting summaries, editorials, letters, published online, reviews, conference proceedings, news, and retracted publications were excluded. All articles published between 2000 and 2021 that met the inclusion criteria were analyzed. Key information from each included article, including title, authors (and their affiliated institutions), abstract, keywords, references, journal name, country/region, and publication year, was downloaded and exported in plain text (TXT) format (Figure 1).

### Bibliometric and visual analysis

Bibliometrix, a scientific bibliometric software program based on R, was used for the bibliometric analysis. The TXT files were imported into Bibliometrix for further data processing. We aim to study the number of articles published annually, their distribution across various countries and regions, the total citation frequency, the H-index, which institutions were the most popular for research in this field, and to analyze the basic characteristics of the data. Bibliometrix, CiteSpace, and VOSviewer software were used to visually display and analyze the collected literature. Through the intuitive display of the coupling and co-occurrence analyses, we were able to understand the status of research in this field and the trends in the development of related fields.

## Results

### Quantitative global publication trends

**Figure 2:**
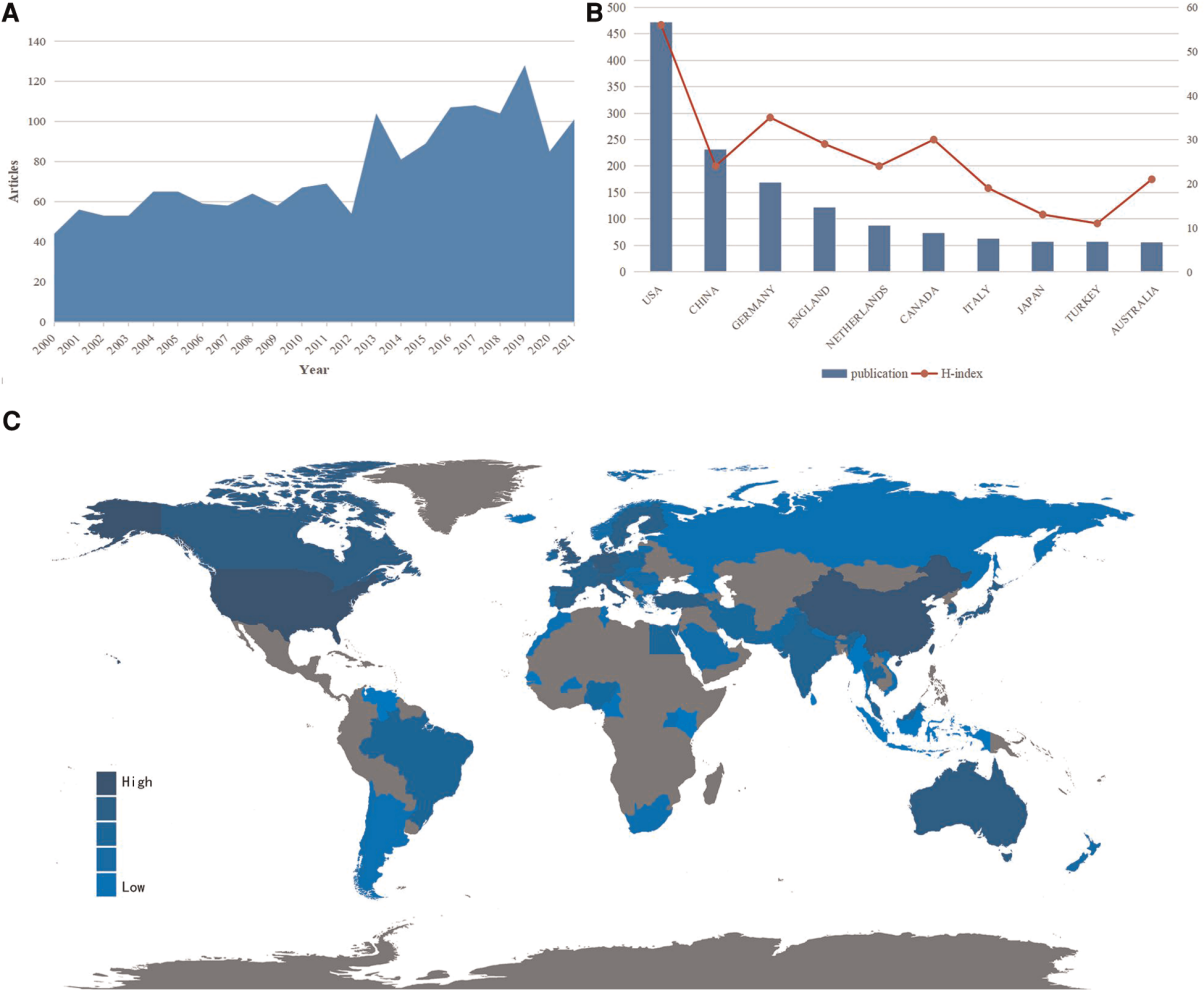
Overview of publications and trends in the field of calcaneal fracture research. (**A**) The number of articles in the calcaneal fracture field published every year between 2000 and 2021. (**B**) The top 10 countries and H-indexes in the field of calcaneal fracture research. (**C**) World map showing the global distribution of calcaneal fracture research; the different colors represent different densities.

A total of 1,687 publications (comprising 1,557 original articles and 130 reviews) that met the selection criteria were identified. The major trends are presented in Figure 2A. Over all, publications on calcaneal fracture research are increasing worldwide. The number of articles published each year increased from 44 (2000) to 101 (2021), peaking in 2019 with 128 articles (representing 7.6% of the total). This suggests that a growing number of scholars are interested in this topic. The map in Figure 2C shows the geographical distribution of these articles.

### Countries and regions of included publications

**Figure 3:**
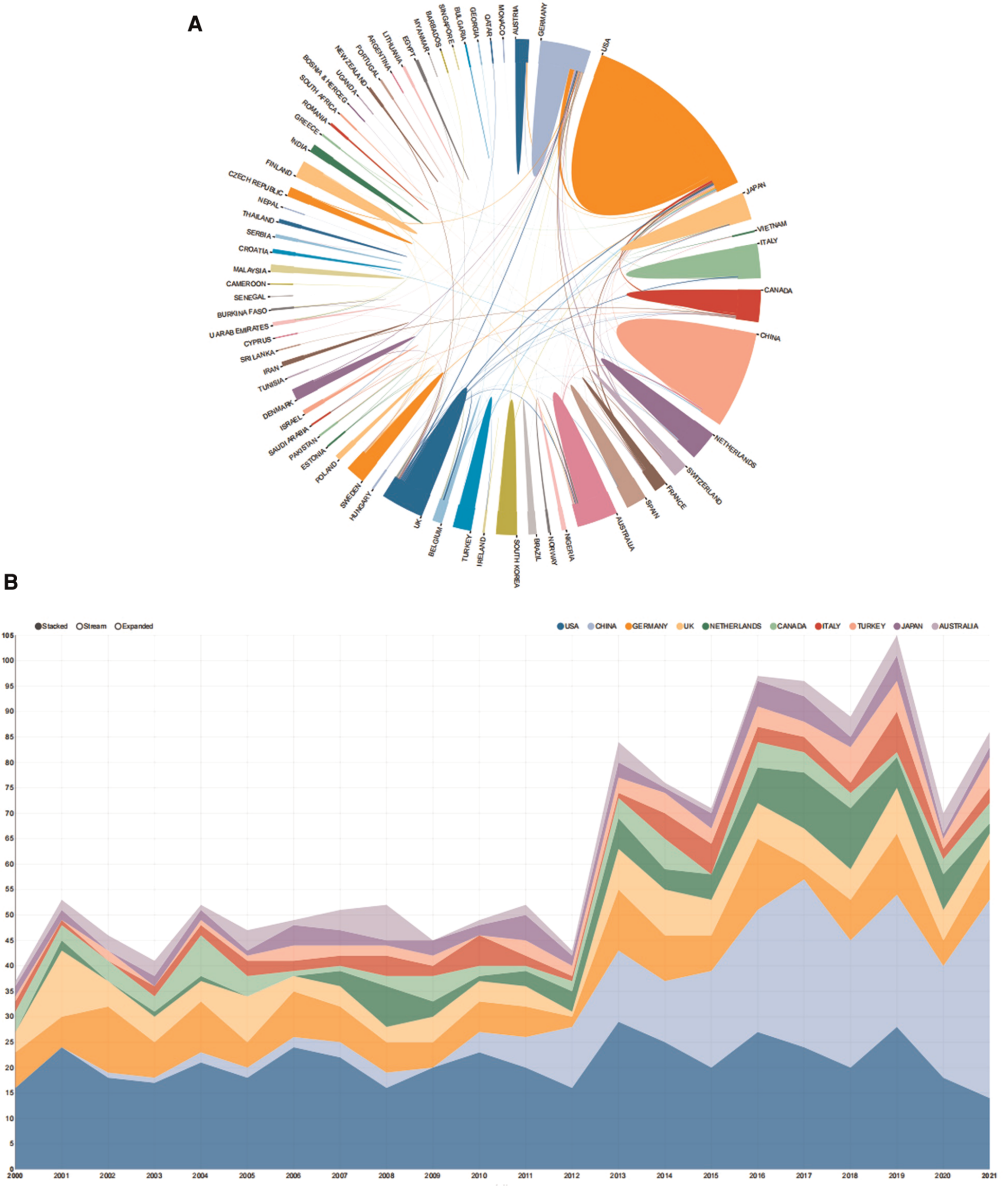
The top countries contributing to calcaneal fracture research. (**A**) Cooperation map of the countries and regions involved in calcaneal fracture research. The colored areas indicate the number of articles published by each country or region. The larger the area, the greater the number of articles. The lines represent the collaborative relationships between countries; thicker lines indicate a closer relationship. This map was generated from an online analysis platform (https://bibliometric.com). (**B**) Publication trends of the 10 countries most active in calcaneal fracture research between 2000 and 2021. The width of each colored area reflects the changes in publication trends among the different countries across the years.

The included articles represent at least 59 different countries and regions. The United States had the largest number of publications on calcaneal fractures, with 471 articles published (27.9% of the total), followed by China (231, 13.7%), Germany (169, 9%), and the United Kingdom (122, 10.0%). As shown in Figure 2B, the United States also had the highest H-index (56). Germany ranked second (35), and Canada ranked third (30). The geographical distribution map (Figure 2C) confirms that articles on calcaneal fracture have been published in most areas of the world. Figure 3B depicts the publication trends in the 10 most active countries in calcaneal fracture research between 2000 and 2021. An analysis of international collaboration among calcaneal fracture researchers showed that the United States collaborated closely with the United Kingdom, Germany, Canada, and Italy (Figure 3A).

**Figure 4:**
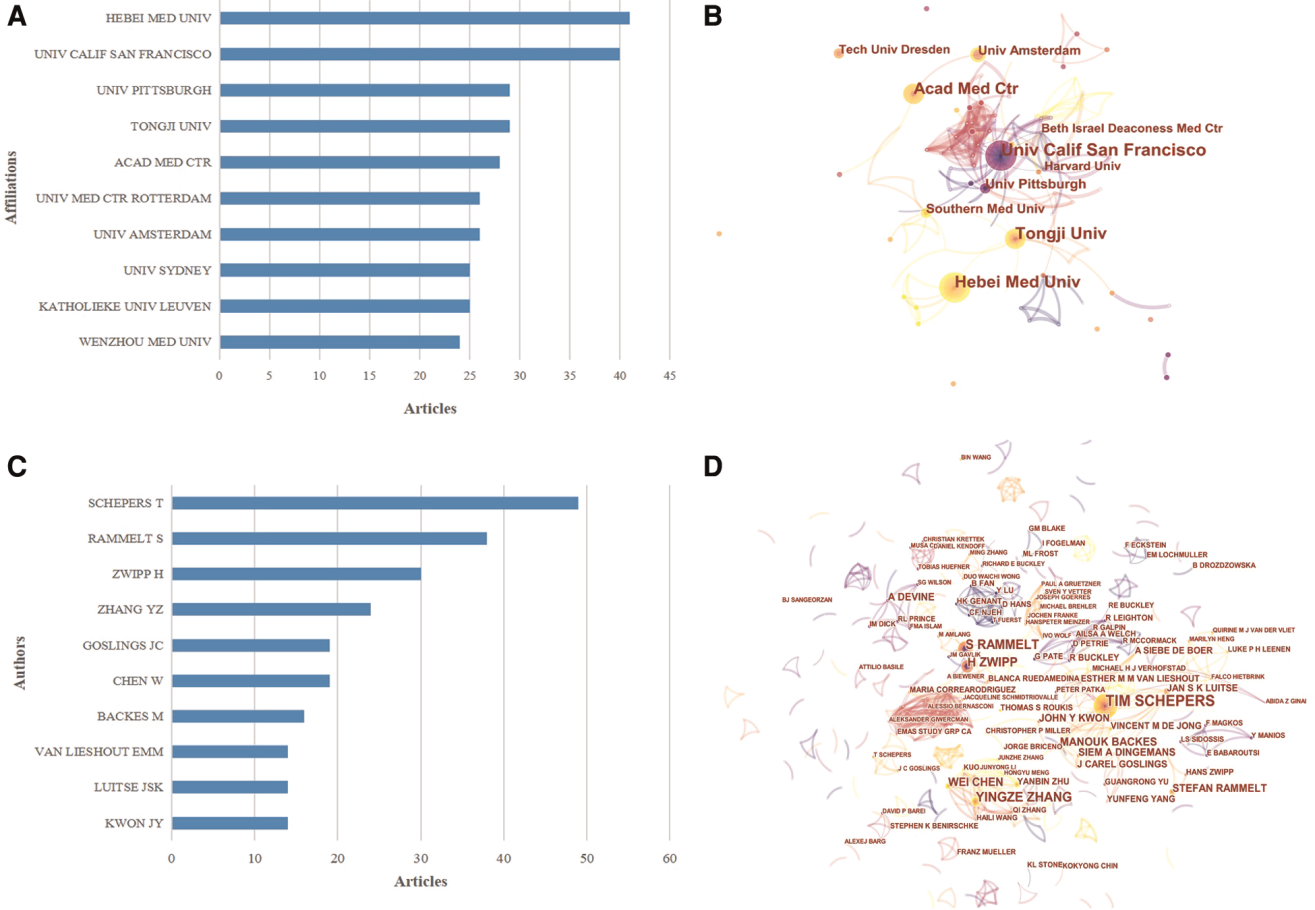
The top 10 authors and institutions contributing to calcaneal fracture research. (**A**) The 10 most relevant affiliations for publications related to calcaneal fracture research. (**B**) Cooperation network map of institutions involved in calcaneal fracture research (generated by CiteSpace). The nodes represent each analyzed object; the greater the frequency of involvement, the larger the node. The color and thickness in the inner circle of the node indicate the occurrence or citation frequency of different time periods. The edge between the nodes and their thickness represent the relationship and strength of co-occurrence or co-citation. (**C**) The top 10 authors and (**D**) the network map of co-cited authors related to calcaneal fracture research. (**D**) Cluster view map of co-cited authors. In the cluster map, cited authors in similar categories are gathered into clusters. Each cluster is labeled with a different color, and the links between nodes represent elements that were cited together. Both maps were generated by CiteSpace.

Contributing institutions As shown in Figure 4A, China’s Hebei Medical University produced the greatest number of publications (41), followed by the University of California, San Francisco (40) and the University of Tongji and University of Pittsburgh (29 each). Figure 4B presents the cooperation visualization map generated by CiteSpace, illustrating the interinstitutional collaborations among the calcaneal fracture research network. This type of collaboration was relatively low, and mainly took place in European and American institutions. The University of California, San Francisco, occupies the center of the collaboration network. Hebei Medical University, the University of California, San Francisco, and the University of Tongji had a BC value > 0.02.

Author contributions The top 10 authors, institutions, and co-cited authors with the largest number of publications are presented in Figure 4. Collectively, these authors have published 1,687 articles over the past 22 years. With the minimum number of citations set to 5 when performing the coupling analysis, a total of 56 authors were ultimately included. The most productive authors came from East Asia, North America, and western Europe. T. Schepers published the highest number of articles (49, i.e., 2.9% of the total), followed by S. Rammelt (38, 2.3%), H. Zwipp (30, 1.8%), and Y. Z. Zhang (24, 1.4%). These four scholars have surely achieved a great deal of expertise in the field of calcaneal fracture research. A list of the top 10 most cited articles (Table 1) and the resulting network of authors was generated by CiteSpace. The network map showed that international collaborative teams were largely disconnected, with less communication between different research institutions. As shown in Figure 4D (a co-citation analysis), T. Schepers had the largest centrality value and greatest number of citations, ranking first among the top 10 co-cited authors.

**Table 1:** Analysis of the top 10 cited articles in the field of calcaneal fracture.

Rank	Article	Journal	Citations	Journal impact factor
1	Buckley et al. (2002)	*Journal of Bone and Joint Surgery*(American Volume)	518	6.558
2	Rammelt et al. (2004)	*Injury*	211	2.687
3	Howard et al. (2003)	*Journal of Orthopaedic Trauma*	172	2.884
4	Harvey et al. (2001)	*Foot & Ankle International*	165	3.569
5	Benirschke et al. (2004)	*Journal of Orthopaedic Trauma*	164	2.884
6	Griffin et al. (2014)	*BMJ—British Medical Journal*	155	93.333
7	Kline et al. (2014)	*Foot & Ankle International*	140	3.569
8	Rammelt et al. (2010)	*Clinical Orthopaedics and Related Research*	135	4.755
9	Al-Mudhaffar et al. (2000)	*Injury—*	126	2.687
10	Agren et al. (2013)	*Journal of Bone and Joint Surgery* (American Volume)	122	6.558

### Journals publishing research on calcaneal fracture

**Figure 5:**
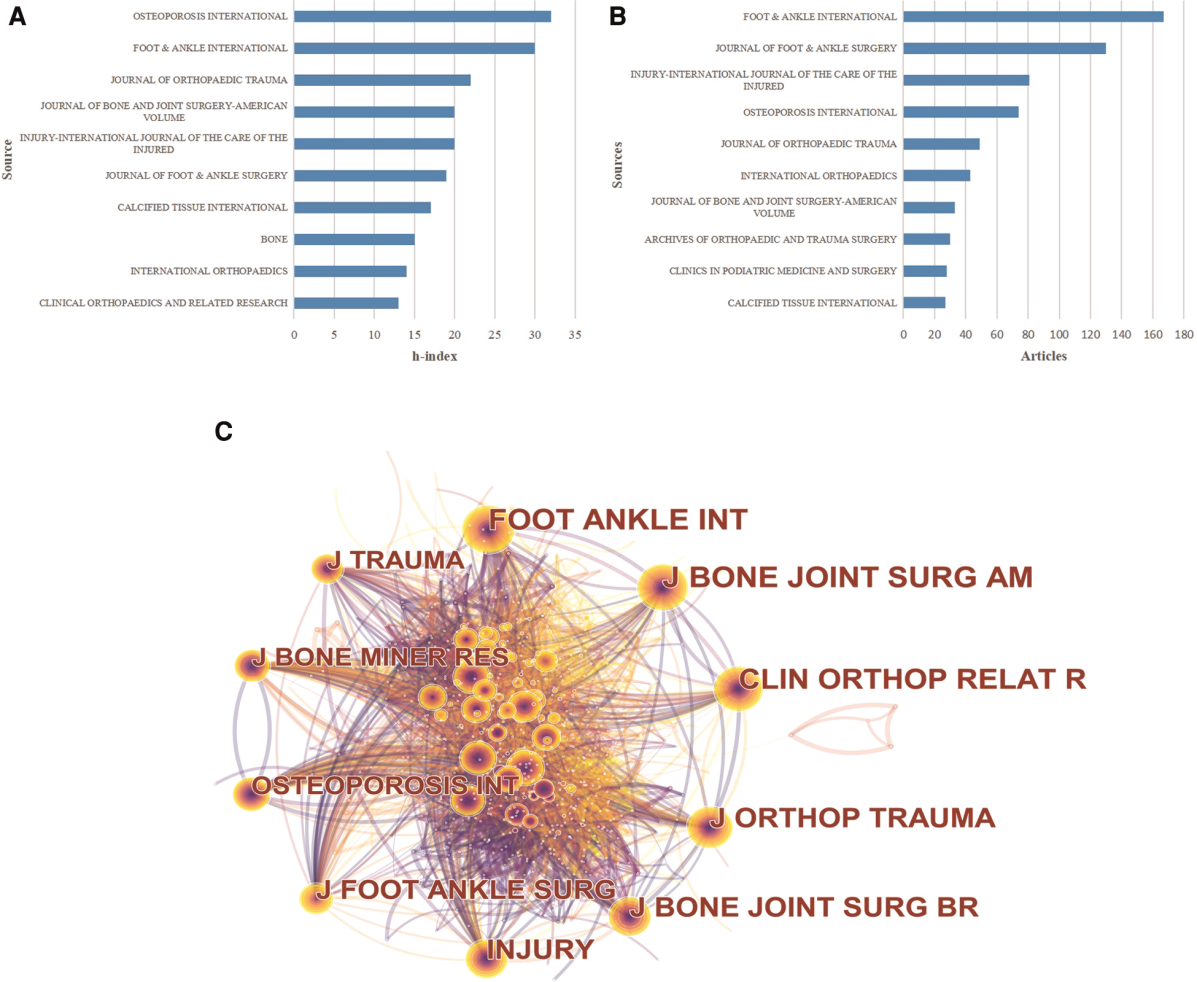
The distribution of journals publishing research on calcaneal fractures. (**A**) The top 10 journals publishing research in the field of calcaneal fracture, ranked by H-index. (**B**) The top 10 journals publishing research in the field of calcaneal fracture, ranked by article count. **C**) Network map of co-cited journals (generated by VOSviewer). Each node represents a different journal, and the node size is proportional to the number of citations; the larger the node, the greater the citation frequency.

A total of 374 journals contain articles on calcaneal fracture. The 10 most popular of these journals have published 662 papers on calcaneal fracture, accounting for 36.24% of the total 1,687 publications. The top 10 journals according to the number of calcaneal fracture publications are shown in Figure 5B. *Foot & Ankle International* published the greatest number of articles (167). *The Journal of Foot and Ankle Surgery* ranked second, with 130 publications, followed by *Injury* (81) and *Osteoporosis International* (74). As shown in Figure 5A, *Osteoporosis International* has the highest H-index value (13), accounting for more than 68.28% of the top 10 journals. In the network map of co-cited journals (Figure 5C), the size of the nodes is proportional to the number of publications published by the journals. Of these, the top 5 journals were *Foot & Ankle International*, the *Journal of Bone and Joint Surgery (American Volume)*, *Clinical Orthopaedics and Related Research*, the *Journal of Orthopaedic Trauma*, and the *Journal of Bone and Joint Surgery (British Volume)*.

### Keywords analysis of research hotspots

**Figure 6:**
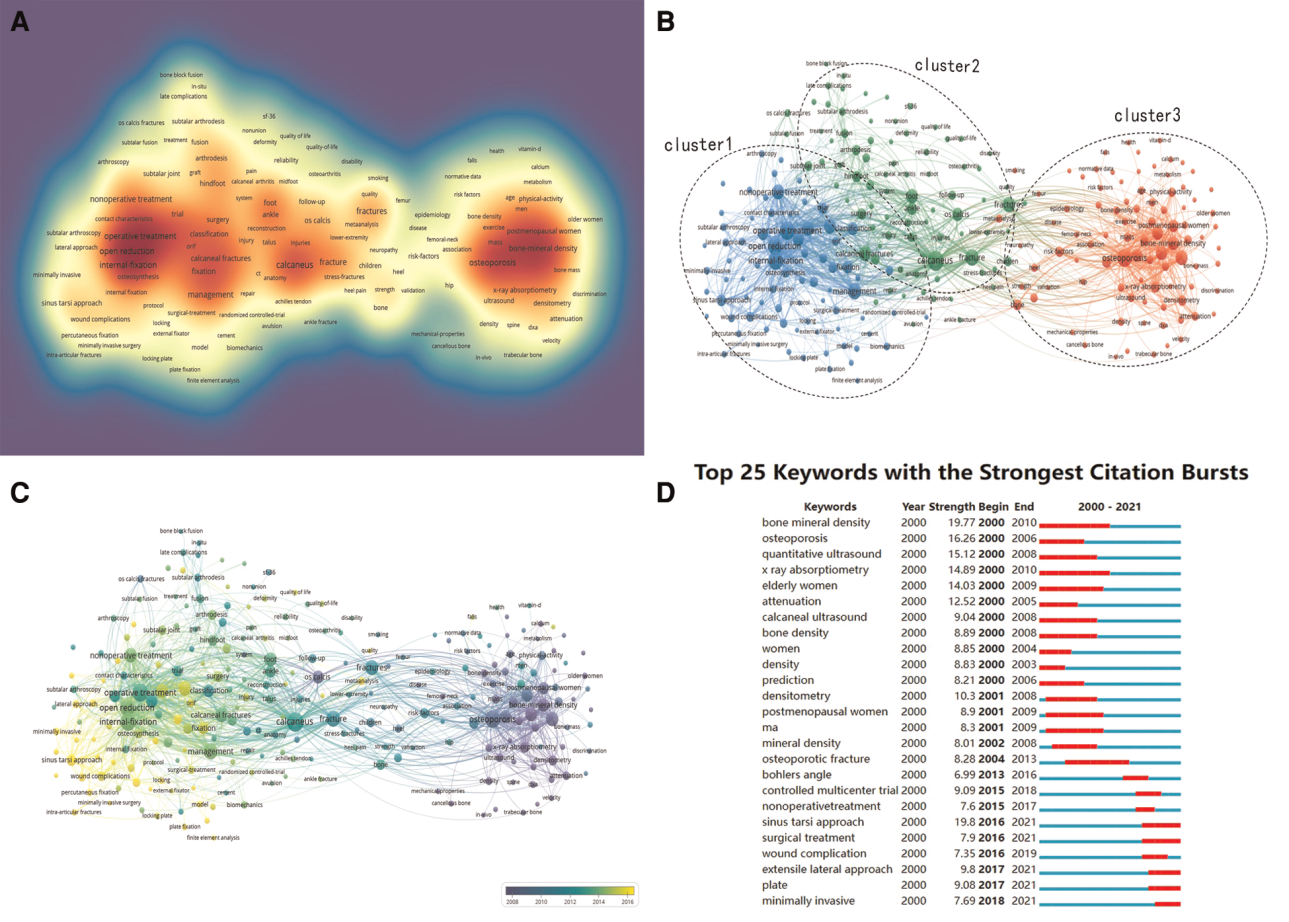
Co-occurrence analysis of global calcaneal fracture research. (**A**) Density map of the keyword co-occurrence analysis (generated by VOSviewer). The darker the color, the higher the keyword density. (**B**) Keyword analysis of research hotspots. Network visualization of the keyword co-occurrence analysis (generated by VOSviewer). (**C**) Overlay map of the keyword co-occurrence analysis using VOSviewer. (**D**) The 25 keywords with the strongest citation bursts between 2000 and 2021. The blue line indicates the time interval, and the red line represents the duration of the keyword’s strongest citation burst.

For the co-occurrence analysis, a total of 4,698 keywords were extracted from the 1,687 publications and analyzed by VOSviewer. As illustrated in the density visualization map in Figure 6A, 250 keywords with a minimum number 10 co-occurrences were included in the analysis, revealing several “hotspot” clusters related to internal fixation, operative treatment, management, and osteoporosis. As shown in Figure 6B, we further classified all co-occurrence keywords into into three clusters: study of internal fixation, study of fracture, and study of osteoporosis. Within the internal fixation cluster, the most frequent keywords were management, internal fixation (occurrence = 297), operative treatment (276), open reduction (232), and nonoperative treatment (164). This cluster was the largest of the three. Within the fracture cluster, the most frequent keywords were foot (occurrence = 136), hindfoot (66), fractures (136), os calcis (117), calcaneal fractures (89), quality of life (136), pain, and stress fractures. Within the osteoporosis cluster, the most frequent keywords were osteoporosis (occurrence = 148), bone mineral density (139), postmenopausal women (91), mineral density (126), x-ray absorptiometry (82), and risk factors (83). Overlay visualization maps can imply co-occurrence, especially those that feature items denoted by color according to the average time period when the keywords occurred (9). In our map (Figure 6C), yellow nodes represent keywords that appeared later, whereas blue represents keywords that appeared earlier. In CiteSpace, “burst” keywords are highly important indicators that reflect the emerging trends and frontiers of research within a specific field (9). Keywords with the strongest citation bursts have, therefore, attracted the greatest attention over a given period of time (9). The top 25 keywords with the strongest citation bursts between 2000 and 2021 are presented in Figure 6D. Notably, several of them have citation bursts that continue into 2021: sinus tarsi approach (2016–2021), surgical treatment (2016–2021), extensile lateral approach (2017–2021), plate (2017–2021), and minimally invasive (2018–2021).

## Discussion

Our bibliometric and visual analysis of the global research output in the field of calcaneal fracture shows an increasing number of publications over the 22 included year. The study of calcaneal fracture has been widespread. However, the overall trend was unclear, and many topics concerning calcaneal fractures remain controversial, and there has been no analysis to date of the “hotspots” in the research field. Therefore, we sorted out several specific aspects to focus on, against the backdrop of global trends in this field, so as to explore the hotspots and emerging frontiers in calcaneal fracture research.

In terms of geographic and institutional distribution, developed countries in the Americas and Europe contributed the largest number of publications in the field of calcaneal fracture research, with China ranking second. The United States, China, Germany, the United Kingdom, and the Netherlands were the top 5 countries in terms of the number of articles published and their centrality. The United States dominated the field, having the greatest number of publications, the highest H-index, and the most citations. This success is likely due to its favorable conditions for basic and clinical medical study, which include advanced equipment, sufficient funds, and a high number of professional researchers (14–20). We could come to the conclusion that researchers in the United States have more advantages than researchers in other countries. However, the performance of two institutions from China (Hebei Medical University and the University of Tongji) was also commendable. Over all, the strength of scientific research from China still needs further improvement. In terms of interinstitutional collaboration, we can see from the cooperation visualization map (Figure 4B) that it remains relatively low. Interinstitutional collaboration was seen mainly in European and American institutions. There are several potential reasons for this: European and American institutions seem to have ideal conditions for basic and clinical medical research, including readily available funds and frequent professional communication among researchers at different institutions. These conditions are frequently noted in similar studies. These regions also have a lot of international conferences, which may be another advantage for their authors. Communication and cooperation between institutions in different countries should be enhanced.

In terms of the author analysis, T. Schepers, S. Rammelt, and H. Zwipp have published the greatest number of articles in this field (Figure 4). Professor T. Schepers has led several biomechanical/cadaver studies and accumulated a great deal of clinical research data on the correlation between the mechanism of injury and fracture classification in calcaneal fracture (21). S. Rammelt and H. Zwipp are associated with the Trauma Department of the University Hospital of Dresden in Germany. They have published many papers on the treatment of calcaneal fracture, on topics such as whether, when, and how to operate on calcaneal fractures (22), open reduction and internal fixation (23), and less-invasive reduction and fixation via a sinus tarsi approach (24). They and their institution provide great guiding value in the area of treatment development for calcaneal fracture, and they have made a great deal of progress in this field. Of note also were the experts from China who are emerging into this field and find themselves on the frontline of calcaneal fracture research, particularly Y.Z. Zhang and W. Chen. W. Chen, in particular, focuses on deep vein thrombosis in isolated calcaneal fracture (25, 26). In general, our research shows that these authors, with their substantial contributions, have played a crucial role in calcaneal fracture research and laid the foundation for future developments in this field.

In terms of journal analysis, *Foot & Ankle International* has published 167 papers on calcaneal fracture, far more than other journals. This journal mainly focuses on foot and ankle surgery. *Foot & Ankle International*, the *Journal of Foot and Ankle Surgery*, *Injury*,*—* and *Osteoporosis International* accounted for more than 68% of all articles in the top 10 journals. They were thus the primary journals for calcaneal fracture research, providing vital information for the field. These results also suggest that publications on calcaneal fracture are more likely to be published in these journals.

The study of the treatment, management, and complications of calcaneal fracture has attracted much attention over the past 22 years. Analyzing the keywords used with these studies can provide insights into the developmental trends of this field, and examining the “citation bursts” can reveal the avenues of future study with the greatest potential value (19). The keywords used, and the research “hotspots” connected to them, have differed at various stages throughout the past 22 years. Before 2010, keywords focused mainly on osteoporosis-related topics, such as bone mineral density, postmenopausal women, x-ray absorptiometry, risk factors, and trabecular bone. We can also see that, over time, researchers paid more attention to the management of calcaneal fracture. In the later stage (between 2016 and 2021), keywords related to the treatment of calcaneal fracture and its complications (such as internal fixation, surgical treatment, plate, sinus tarsi approach, wound complications, minimally invasive technique, and extensile lateral approach) have become much more prominent. As is widely known, open reduction and internal fixation (ORIF) has become the gold-standard treatment for calcaneal fracture. However, an increasing number of articles are reporting complications of the surgical wound. A study by Jianming Chen that presented a minimally invasive internal fixation through a small tarsal sinus incision has attracted a lot of attention in recent years (27). His study indicated that percutaneous techniques, in sinus tarsi versus extensile lateral approaches, and a minimally invasive technique with a locking plate can improve or mostly restore the Böhler and/or Gissane angles, with a mean follow-up of approximately 42 months (27). Martin S. Davey presented a similar result with the sinus tarsi approach in 58 patients with an increased risk of complications due to smoking (28). With a growing number of keywords on the minimally invasive treatment of calcaneus fixations, this surgical method seems to be a major trend. A study by H. Wang found that the wound infection rate in closed calcaneus fractures was between 2% and 25% (29). T. Schepers conducted a systematic review of open calcaneal fracture and found their complication rates to be higher than those of closed fractures (30). Complications involving surgical wounds, including hematoma, wound infection, wound breakdown, and skin edge necrosis, are a major concern (1). A risk prediction model for calcaneus fracture would help to provide early warning regarding wound complications (31). A study by K.E. Spierings showed that it was wounds treated through the sinus tarsi approach have a lower risk of complications (13). His study confirms the low risk of SSI in DIACFs treated via STA. Significant predictors for SSIs were surgery within one week after injury, ASA of 2 or higher and blood loss >150cc.

Reducing wound complications has become a focus of research. A prospective randomized clinical trial (32) to compare the functional and radiological outcomes of the sinus tarsi and extended lateral approaches for calcaneal fractures found broadly similar results between the two, though the sinus tarsi approach demonstrated the potential to decrease preoperative waiting times and wound complication rates. There is abundant evidence from cadaveric studies to suggest that the sinus tarsi approach is safe (33, 34). A. Herlyn reported that a new interlocking nail, used with a minimally invasive locking nail technique, could reduce complications and achieve superior functional outcomes compared to standard locking plates (35). This calcanail system (C-nail) represents a novel intramedullary approach for calcaneal fractures. A finite -element analysis showed that the modified C-nail system can provide comparatively sufficient stability, making it preferable for the treatment of complex calcaneal fracture (36). A comparative retrospective study reported that the C-nail may be a successful alternative in the treatment of calcaneal fracture because of the minimally invasive implantation and high primary stability (37). The outcomes obtained with C-nail fixation are statistically identical to those obtained with locking calcaneal plate (LCP) fixation. LCP fixation and C-nail fixation had the same outcomes in the treatment of calcaneal fractures of Sanders types II and III (38). The minimally invasive dual incision and internal fixation with a mini-plate may be effective in terms of radiological outcomes and offer a lower rate of wound infections (39). However, in recent years, the continuous development of implants, including screws, nails, and plates (40), as well as new techniques, especially in 3D printed models (41), and biomechanical studies (42), may contribute to improving patient outcomes after surgery. The evolutionary trends of the keywords used in calcaneal fracture research are shown in Figure 6. Keywords with the latest average appearing year will help us understand and master the trends and future research hotspots. These studies have received much attention recently, and further in-depth studies are required.

## Limitations

Although our study is the first ever to conduct a bibliometric analysis of peer-reviewed papers related to calcaneal fracture, it still has several limitations, which we aim to improve in the future. First, the data from our study came only from WOSCC and focus only on the years 2000–2021. As the database is always being updated, limitations of software and analytical methods may come into play. Second, our study included only English-language articles; therefore, high-quality work in other languages, such as Chinese, was not part of our analysis.

## Conclusion

The United States has made outstanding contributions in the field of calcaneal fracture research, and Professor T. Schepers has made the greatest number of individual contributions. calcaneal fracture field. The most prolific institution for calcaneal fracture research was Hebei Medical University. The “hotspots” of research have shifted over time from disease management to prevention. We recommend that more attention should be paid to the following research trends: the sinus tarsi approach, wound complications, minimally invasive techniques, the extensile lateral approach, surgical treatment, and implants. Ultimately, this study reveals the current hotspots and frontiers of calcaneal fracture research, and these considerations can inform various levels of practice and future decision-making.

## Data Availability

The original contributions presented in the study are included in the article/Supplementary Material; further inquiries can be directed to the corresponding author/s.
